# Machine Learning Models for Pancreatic Cancer Survival Prediction: A Multi-Model Analysis Across Stages and Treatments Using the Surveillance, Epidemiology, and End Results (SEER) Database

**DOI:** 10.3390/jcm14134686

**Published:** 2025-07-02

**Authors:** Aditya Chakraborty, Mohan D. Pant

**Affiliations:** Department of Epidemiology, Biostatistics, and Environmental Health, Joint School of Public Health, Old Dominion University, Norfolk, VA 23529, USA; pantmd@odu.edu

**Keywords:** SEER Database, pancreatic cancer, cancer disparities, goodness of fit, parametric survival analysis, predictive survival models

## Abstract

**Background:** Pancreatic cancer is among the most lethal malignancies, with poor prognosis and limited survival despite treatment advances. Accurate survival modeling is critical for prognostication and clinical decision-making. This study had three primary aims: (1) to determine the best-fitting survival distribution among patients diagnosed and deceased from pancreatic cancer across stages and treatment types; (2) to construct and compare predictive risk classification models; and (3) to evaluate survival probabilities using parametric, semi-parametric, non-parametric, machine learning, and deep learning methods for Stage IV patients receiving both chemotherapy and radiation. **Methods:** Using data from the SEER database, parametric models (Generalized Extreme Value, Generalized Pareto, Log-Pearson 3), semi-parametric (Cox), and non-parametric (Kaplan–Meier) methods were compared with four machine learning models (gradient boosting, neural network, elastic net, and random forest). Survival probability heatmaps were constructed, and six classification models were developed for risk stratification. ROC curves, accuracy, and goodness-of-fit tests were used for model validation. Statistical tests included Kruskal–Wallis, pairwise Wilcoxon, and chi-square. **Results:** Generalized Extreme Value (GEV) was found to be the best-fitting distribution in most of the scenarios. Stage-specific survival differences were statistically significant. The highest predictive accuracy (AUC: 0.947; accuracy: 56.8%) was observed in patients receiving both chemotherapy and radiation. The gradient boosting model predicted the most optimistic survival, while random forest showed a sharp decline after 15 months. **Conclusions:** This study emphasizes the importance of selecting appropriate analytical models for survival prediction and risk classification. Adopting these innovations, with the help of advanced machine learning and deep learning models, can enhance patient outcomes and advance precision medicine initiatives.

## 1. Background

Pancreatic cancer continues to be one of the significant health hazards affecting people all over the globe. Due to its high lethality, pancreatic cancer shows almost the same rates for incidence and mortality [1]. According to recent findings, this illness is responsible for nearly 30,000 deaths per year in the U.S. [2]. It is the fourth principal reason for cancer deaths in the USA and leads to an estimated 227,000 deaths per year worldwide. The incidence and number of fatalities from pancreatic tumors have been continuously increasing, while the incidence and mortality from other prevalent cancers have been decreasing. Despite advancements in pancreatic cancer detection and care, it is estimated that only approximately 4% of patients will survive five years following diagnosis [3]. Although in most cases pancreatic cancer remains incurable, researchers have focused on how to enhance the survival rates of individuals with pancreatic cancer via statistical modeling [4,5,6]. Advancements in the molecular understanding of pancreatic cancer have enhanced our knowledge of the intricate genetic and metabolic changes that propel tumor progression. These insights have broadened the scope of potential therapeutic targets and clinical management strategies, yet substantial challenges persist in converting these discoveries into tangible improvements in patient outcomes. Disparities in access to high-quality cancer care and treatment have been observed in pancreatic cancer, particularly among patients from lower socioeconomic backgrounds and diverse racial/ethnic groups. Implementing strategies to address these disparities, such as enhancing early detection, improving access to multidisciplinary care, and increasing enrollment in clinical trials, may help to ameliorate outcomes for underserved patient populations [7]. As the field of precision medicine continues to evolve, the integration of various data sources, including clinical, genomic, and imaging, is becoming increasingly important for accurately predicting the prognosis of pancreatic cancer patients [8,9]. Survival analysis combined with parametric and predictive modeling approaches can play a crucial role in this endeavor, providing a robust framework for modeling the time-to-event outcomes and identifying the factors that influence the survival of these patients. Integrating survival analysis into parametric and predictive modeling approaches can significantly enhance the accuracy and personalization of patient outcome predictions [10]. Parametric survival models assume that the time-to-event variable follows a specific probability distribution, such as exponential, Weibull, or log-normal, allowing for more precise estimation of survival probabilities and hazard rates when the assumption is met [11]. Additionally, predictive modeling techniques, including machine learning algorithms, can learn complex patterns from high-dimensional data, capturing non-linear relationships and interactions between variables that traditional methods may overlook. By combining survival analysis with parametric and predictive modeling approaches, clinicians and researchers can gain a more comprehensive understanding of the factors influencing patient outcomes, leading to more informed decision-making and personalized treatment strategies.

In this study, a comparison was made across seven different models for computing the survival probabilities at different time points. Four of the seven models were widely used predictive models (Gradient Boosted Machine (GBM), elastic net (EN), random forest (RF), and neural network (NN)), and the remaining three were the traditional Kaplan–Meier (KM), Cox Proportional Hazard (CPH), and the parametric survival models. One of the most important goals was to check how robust the predictive models were in comparison with the Kaplan–Meier, Cox PH, and the parametric survival models.

## 2. Materials and Methods

### 2.1. Study Setting and Data Description

**Figure 1:**
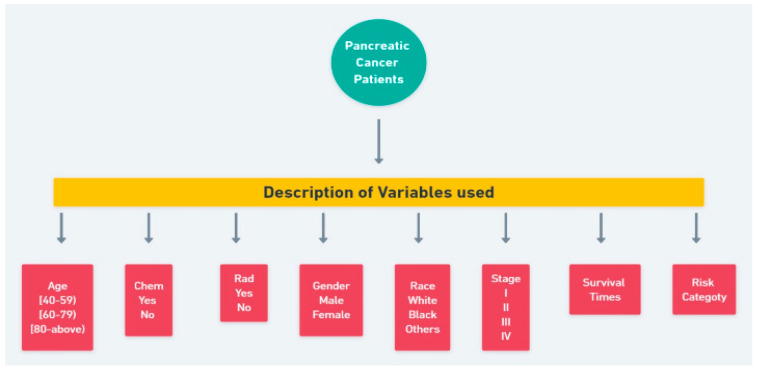
Description of study variables.

The present study uses data from the Surveillance, Epidemiology, and End Results (SEER) database (https://seer.cancer.gov/) accessed on 25 January 2025, which contains information on patients diagnosed with pancreatic adenocarcinoma. The data were extracted using the SEER*stat software version 8.4.5 (https://seer.cancer.gov/seerstat/) accessed on 25 January 2025. The analysis was based on the survival times (in months) as the outcome and cause-specific death (deaths due to pancreatic cancer) for each patient. The survival times of patients are one of the most pivotal factors used in all cancer research. It is necessary to evaluate the severity of cancer, which helps to determine the prognosis and identify the correct treatment options. The sample size for the study was 5000. Data on patients diagnosed with and deceased from pancreatic adenocarcinoma were extracted for analysis, stratified by race (White, Black, and Other) and cancer stage. The dataset also included information on treatment options, including chemotherapy (C) alone, radiation (R) therapy alone, and a combination of both treatment types (C + R). The age variable was partitioned into three different groups: [40–59), [60–79), and [80–above). The outcome variable (survival time) was divided into three risk groups; $R_{1}:\;[0-4.2],\;R_{2}:\;(4.2-12.6]$, and $R_{3}:\;(12.6-138.6].$ The schematic diagram of the data used in this study on the different races, cancer stages, and age groups is shown in Figure 1. Since most pancreatic cancer cases develop at or after the age of 45, we excluded patients younger than 40 [12]. One of the initial goals of our study was to improve survival probability estimates by identifying the most appropriate probability distributions that characterize the probabilistic behavior of patient survival times for different patient groups, parametrically, to perform parametric survival analysis [13,14].

Before determining the most accurate probability distribution of survival times, the following questions were answered.

1. If there is any statistically significant difference in median survival times among the race groups [15].
2. If there is any statistically significant difference in median survival times among the age groups.
3. If there is any statistically significant difference in median survival times between the gender groups.
4. If there is any statistically significant difference in median survival times among the cancer stages of patients who underwent only chemotherapy, who underwent only radiation, and who underwent a combination of chemotherapy and radiation.

To answer the above questions, we used the nonparametric Kruskal–Wallis (KW) test [16], as the study data were non-normal (skewed) in nature. If a significant difference was found, then multiple comparison tests were performed, followed by the KW test. The Holm–Bonferroni [17] method was used to compute the adjusted *p*-values for the multiple comparisons. After we answered the above questions, we used goodness-of-fit (GOF) tests [18,19,20] to identify the analytical forms of survival times for different patient groups. Various probability distributions were evaluated against the study data across different patient groups. Among these, the Generalized Extreme Value (GEV) distribution [21] and the Generalized Pareto (GP) distribution [22] were identified as the most appropriate models for distinct patient subgroups and at different cancer stages. All statistical analyses were conducted using a predefined significance level of 0.05.

### 2.2. Developing Predictive Models for Classifying Patients into Risk Categories and Performing Survival Analysis

Developing robust predictive models to classify patients into distinct risk categories is a vital advancement in contemporary healthcare, with the potential to transform patient management, resource utilization, and clinical decision-making [23]. The outcome variable risk, with categories $R_{1}$, $R_{2}$, and $R_{3}$, was used to classify patients based on the predictors. Five popular predictive models were used to perform the task [24]. First, the models were compared across the three treatment categories, and later, the performances of the models were evaluated, irrespective of the treatment categories. Each model was trained using 70% of the dataset, while the remaining 30% of the dataset was reserved as test data for model validation. The accuracies and RMSEs were computed based on the test data. Later, seven different models (Gradient Boosted Machine, elastic net, random forest, neural network, Kaplan–Meier, Cox PH, and parametric Generalized Extreme Value) were constructed for computing the survival probabilities at different time points.

## 3. Results

### 3.1. Hypotheses Testing for Specific Attributes

**Figure 2:**
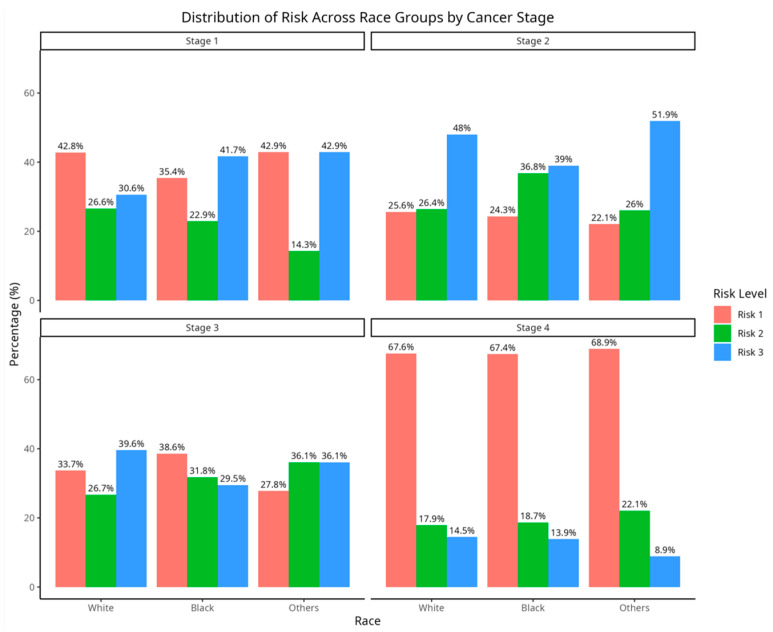
Distribution of risk groups across race and cancer stages.

The following Figure 2 illustrates the distribution of patient risk categories across diverse racial groups and cancer stages. It gives an idea of the data distribution across the stages. Each subplot corresponds to a specific cancer stage and displays the proportion of individuals in each risk level ($R_{i},\;i=1,2,3$) within each racial cohort. In the early stages, the distribution of risk levels exhibits greater variability across racial groups, without a single risk level dominating. However, in more advanced stages, Risk 1 ($R_{1}$) emerges as the predominant category across all racial groups, particularly in Stage 4, where over 67% of individuals in each racial group are classified as $R_{1}$. This trend suggests a shift towards increased representation in the highest risk category (reduced survival times) as the cancer progresses.

Table 1 displays the KW test statistics, along with the *p*-values for race, age, gender, and stages, across the three treatment options (chemotherapy only, radiation only, and a combination of both). No significant differences in survival times were found among the race and gender groups. However, patient survival differed significantly across the age groups. As suggested by the KW test, the three age groups were significantly different from each other. While performing the post hoc tests, across each group, significant difference was found (*p* < 0.0001: age group 1 ([40–59)) vs. age group 2 ([60–79)), *p* < 0.0001: age group 1 ([40–59)) vs. age group 3 ([80-above)), and *p* < 0.0001: age group 2 ([60–79)) and age group 3 ([80-above))). Moreover, a significant difference in patient survival was prominent across the stages for all treatment options. Table 2 shows the results of the post-hoc tests for the following three treatment categories.

From Table 2, we see that no significant difference was found between cancer stages 1 and 2 for all three treatment groups. The difference was significant for Stages 2 and 3 and Stages 3 and 4 for all the groups, except for the group that was administered radiation only. However, for category 2 (radiation only), median survival times differed across cancer Stages 1 and 4 (*p* = 0.022). No significant difference in survival was found across Stage I, Stage II, and Stage III for the patients with radiation only.

### 3.2. Identifying the Analytical Forms of the Survival Times Across the Cancer Stages for Different Treatment Options

The analytical forms (probability distribution functions) of the survival times of the patients at different cancer stages have been computed by estimating the parameters of the distributions from the available data that helped to estimate the Parametric Cancer Survival Probability Estimates (PCPSPs). All probability distributions have been identified by the GOF tests.

Table 3 illustrates the probability distributions by estimating the parameters at different cancer stages. For category 2, where the patients were administered radiation only, the data for Stage I, Stage II, and Stage III were combined, as no significant differences were found among the three cancer stages (Table 2). The combined analytical form (Stage I + Stage II + Stage III) was found to be a Generalized Extreme Value (GEV) probability distribution.

Also, the Generalized Extreme Value (GEV) (with different location (μ), scale (σ), and shape (k) parameters) was found to be the most suitable probability distribution for most of the cancer stages for all the categories.

**Figure 3:**
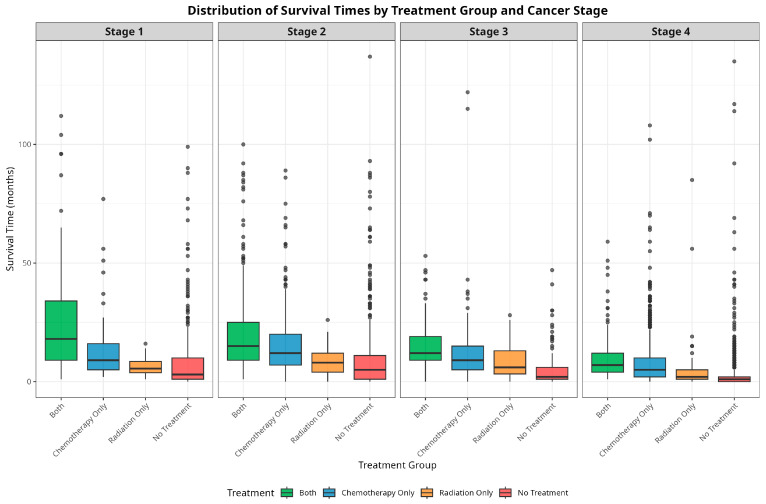
Distribution of survival times by treatment groups across cancer stages.

Figure 3 illustrates the distribution of time-to-death across cancer stages, stratified by the treatment options. When compared to patients who receive only one treatment or no treatment at all, patients who receive both chemotherapy and radiation therapy typically have longer median survival times across all stages.

### 3.3. Classifying Patients into the Risk Groups Using Predictive Models

**Figure 4:**
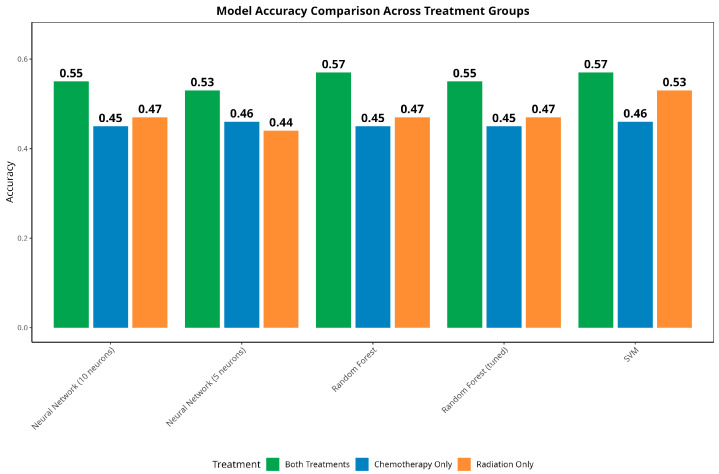
Comparing the performance of the predictive models across the three treatment categories.

**Figure 5:**
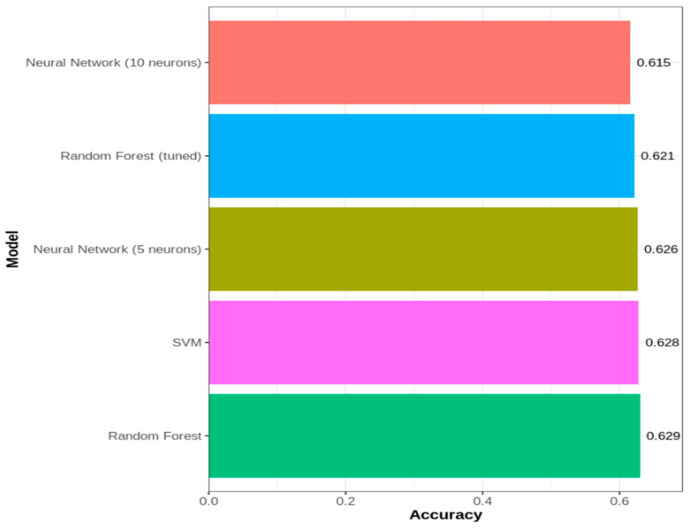
Comparing the overall performances of the predictive models.

To understand the predictive applicability, five different predictive models were developed (70% training data) and validated (30% test data) using the survival data, considering risk as the outcome variable with categories $R_{1},\;R_{2}$, and $R_{3}$, given the other predictors The accuracy of each of the models was based on the test data. Corresponding to each of the three treatment categories (chemotherapy only, radiation only, and combination), five predictive models were tested, resulting in a total of fifteen models (Figure 4). The performances of the models were also compared with the overall dataset, irrespective of the treatment groups (Figure 5).

The analysis of model performance across various treatment groups reveals notable patterns in predictability. Patients receiving both chemotherapy and radiation treatments demonstrate the most predictable outcomes, with random forest achieving the highest accuracy of 57% and the lowest RMSE of 0.826. In contrast, patients receiving only chemotherapy exhibit the least predictable outcomes, with the best model achieving an accuracy of only 46% and an RMSE of 1.072. The radiation-only group falls between these two, with SVM achieving 53% accuracy and an RMSE of 0.816. The predictive accuracies of all five predictive models were found to be consistent across the treatment categories.

In the overall model comparison (irrespective of the treatment group), a similar trend of consistency in the accuracy was noticed.

**Figure 6:**
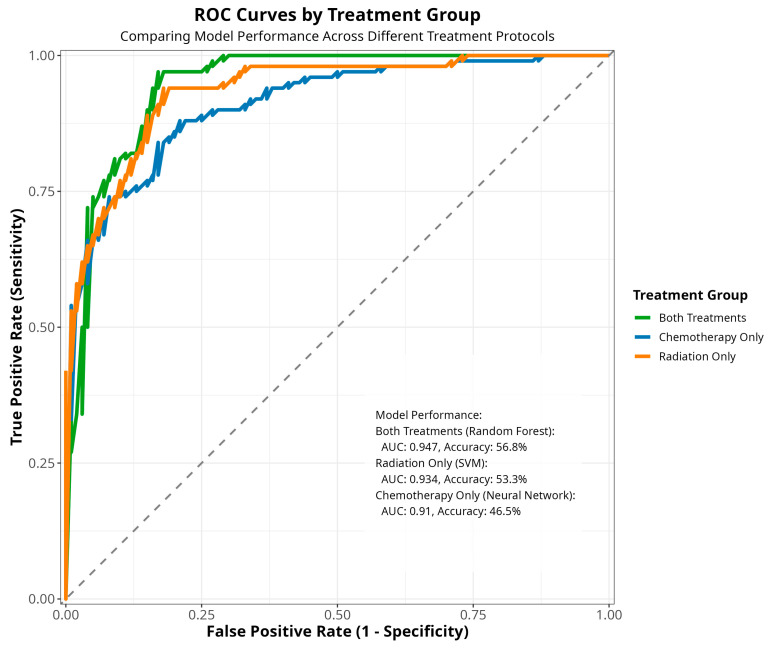
Comparison of the ROC curves across the three different treatment groups.

The ROC curve analysis (Figure 6) further substantiates the differences in model performance across treatment groups. The ‘both treatments’ cohort exhibits the highest area under the curve (AUC: 0.947, accuracy: 56.8%), outperforming the ‘radiation only’ group (AUC: 0.934, accuracy: 53.3%), while the ‘chemotherapy only’ group demonstrates the lowest AUC (AUC: 0.91, accuracy: 46.5%). This consistent pattern observed across multiple evaluation metrics strongly suggests that the type of cancer treatment received is a pivotal factor in determining the predictive accuracy of machine learning models for patient outcomes.

### 3.4. Comparing the Traditional Survival Models with the Predictive Survival Models

Table 4 illustrates the survival probabilities obtained from the seven different models for patients at Stage IV, who have undergone both chemotherapy and radiation $\left(n=228\right).$ The parametric survival probabilities (driven by the GEV (μ=5.7, σ=4.5 , k=0.36) probability distribution that was identified under Section 3.2) were compared with the non-parametric Kaplan–Meier (KM), semi-parametric Cox Proportional Hazard (CPH), and four predictive survival probability estimates [25,26,27] at different time points. Similarly, the probabilities estimated can be computed for any of the patient groups at any specific stage. The median survival time from the GEV was found to be 7 months (95% CI: [6.7, 8]).

**Figure 7:**
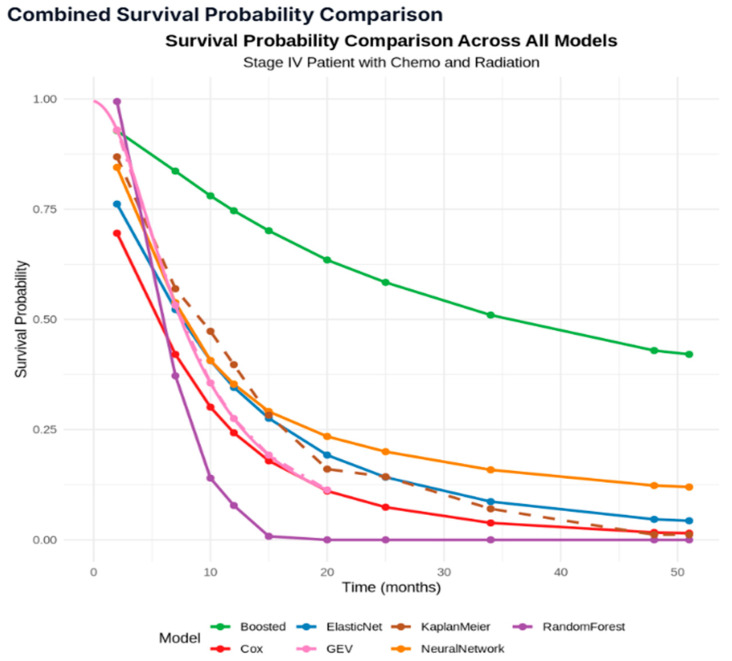
Comparing the survival curves for the seven models.

The survival curves in Figure 7 reveal significant disparities among the seven statistical models in forecasting survival probability for Stage IV cancer patients undergoing a combination of chemotherapy and radiation. The GBM (boosted model) shows the most optimistic survival estimates, maintaining the highest probabilities throughout the entire time period (0.92 at t=2 declining to 0.32 at t=51). A distinctive rapid decline in the survival curve was seen by the random forest (RF) model, predicting near-zero survival probability after approximately t=15. The CPH model shows a steady decline from survival probability 0.70 at t=2 to approximately 0.01 at t=51. The GEV and the CPH models show similar patterns and become almost indistinguishable after t=15. The elastic net (EN) and neural network (NN) also show a similar pattern. However, after t=15, a decreasing trend in the survival curve was observed for the EN model.

**Figure 8:**
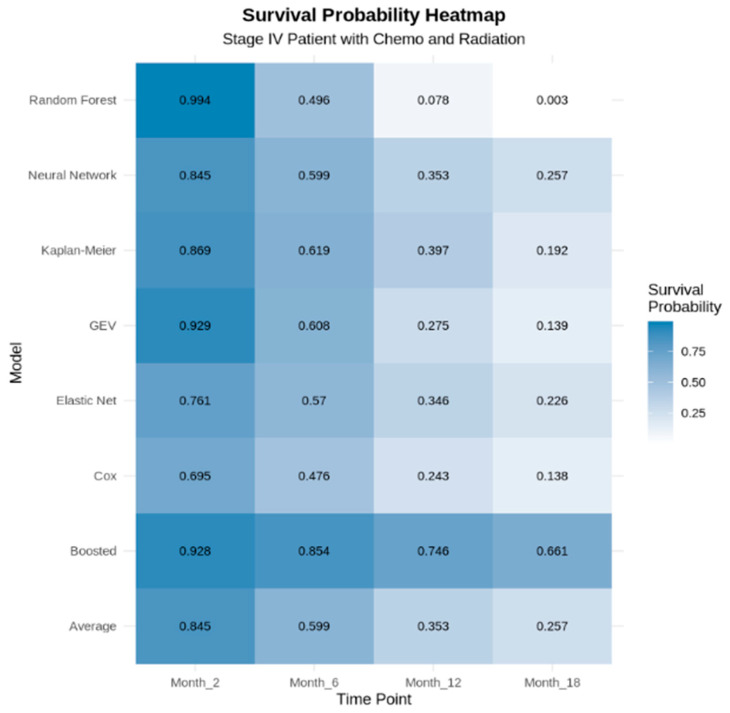
Comparing the survival probabilities at 2, 6, 12, and 18 months for Stage IV patients with chemotherapy and radiation.

Figure 8 illustrates the survival probabilities at 2 months, 6 months, 12 months, and 18 months, respectively, using a heatmap, across all seven models. As shown in Figure 7 and Figure 8, all models exhibited relatively consistent patterns in probability estimation, with the exception of the GBM (boosted), which consistently produced the most optimistic predictions among all models.

**Figure 9:**
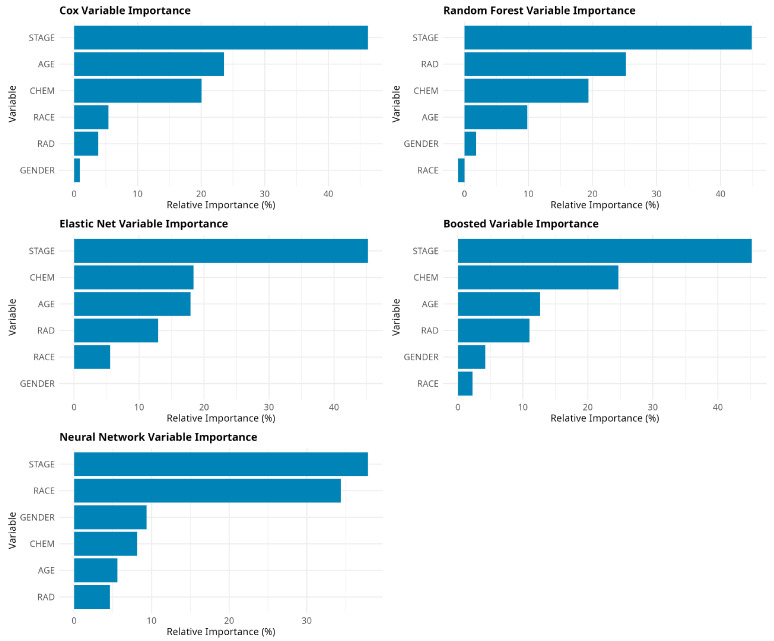
Variable importance (VIP) plots based on different models.

Figure 9 compares the Variable Importance Plots (VIPs) across different models. All the models suggest that the cancer stage is the most important predictor. Age was listed as the second most important predictor by the CPH, the third most important predictor by both the EN and GBM, the fourth most important predictor by the RF, and the fifth most important predictor by the NN. Both the EN and GBM listed chemotherapy as the second most important predictor, and radiation therapy as the fourth most important predictor, whereas both the CPH and RF indicated chemotherapy as the third most important predictor. According to the NN, chemotherapy was the fourth most important predictor. Radiation therapy was the second, fifth, and sixth most important predictors by the RF, CPH, and NN models, respectively. Race and gender were found to be the least important predictors, except for in the NN model.

## 4. Discussion

Combining the parametric, traditional, and machine learning (ML) approaches, this study focuses on performing survival analysis by comparing the survival probability estimates at different time points. The survival times (outcome variable) of patients were compared based on different patient groups, and the analytical forms of the survival times (in months) were identified at each cancer stage parametrically, which were instrumental in computing the Pancreatic Cancer Parametric Survival Probability Estimates (PCPSPs). The parametric survival probability estimates were then compared with the widely used KM and CPH probability estimates, along with four other popular predictive models for Stage IV patients (chemotherapy and radiation) [28]. The intrinsic distributional assumption regarding the survival times under study was one of the difficulties in the parametric survival analysis. However, if one can justify the distributional assumptions via goodness-of-fit tests, it is possible to obtain better estimates from the parametric analysis, which usually produces better statistical power [29]. If additional attributes [15] (risk factors, socio-economic status, and medication) related to the patient’s survival are available from the data source, an analytical model/predictive model [28,30,31,32] can be developed by taking the patient information into account, which can be deployed for further use. The comparative survival study across demographic and clinical factors reveals critical policy implications for addressing disparities in pancreatic cancer outcomes. The observed variations in survival probabilities across racial groups and stages underscore the urgent need for equitable access to early detection, timely diagnosis, and evidence-based treatment. These findings highlight structural barriers in healthcare delivery, including differential access to multimodal therapies, such as surgery and chemotherapy, which disproportionately impact marginalized populations [33]. The current research utilizes a predictive modeling approach to classify individual patients based on three risk classification groups with a certain degree of accuracy. By stratifying patients under each treatment group (chemotherapy, radiation therapy, and combination), and developing five individual predictive models for each treatment group, these findings indicate that the treatment type significantly influences risk predictability, with the combined therapy producing more consistent patterns than the single-treatment approaches, particularly chemotherapy alone (Figure 4). The results obtained from the traditional and the predictive survival comparisons (Figure 7) give us valuable insights regarding the applicability of the models in the estimation of time-to-event outcomes, such as disease recurrence or patient mortality, which supports informed clinical decision-making and resource planning. This stark contrast underscores the pivotal role of model selection in shaping prognostic estimates, and the pronounced variation in the survival curves strongly suggests that clinicians should consider multiple modeling approaches when estimating patient survival, ensuring they avoid overly optimistic or pessimistic prognoses. The ROC curves in Figure 6 provide a clear visual representation of the performance disparities between the three treatment groups, corroborating the earlier findings from the accuracy comparisons (Figure 5). The “both treatments” group exhibits the strongest predictive capabilities with the highest AUC, followed by the “radiation only” group, while the “chemotherapy only” group displays the weakest performance.

Collectively, these consistent patterns across multiple evaluation measures firmly indicate that the treatment type is a critical determinant in the ability to accurately forecast patient outcomes using machine learning approaches. Evaluating machine learning models for survival prediction is crucial, as various algorithms exhibit differing capabilities in handling complex, high-dimensional data, nonlinear relationships, and censored observations [34]. By assessing the predictive performances of contrasting ML models and deep learning (DL) approaches, researchers can identify the most accurate and generalizable tools for specific datasets or clinical settings. This comparative analysis not only enhances the robustness of survival predictions but also facilitates the selection of models that balance interpretability and predictive performance, ultimately improving patient stratification and personalized treatment planning. To improve survival outcomes and reduce inequities, policy efforts should prioritize expanding community-based screening and education programs, enhancing healthcare infrastructure in underserved areas, and promoting the integration of geriatric oncology and precision medicine approaches. Furthermore, resource allocation guided by survival probability estimates can support targeted interventions and survivorship care planning for high-risk subgroups [35]. Addressing the social determinants of health remains essential to mitigating non-clinical barriers to care. Collectively, these policy implications call for a multi-level response that integrates health equity principles into pancreatic cancer prevention, treatment, and survivorship strategies.

## Figures and Tables

**Table 1:** Kruskal–Wallis test results for different categories.

Category	KW Test Statistic	*p*-Value
Race	0.68	0.71
Age	291.50	<2.2 × 10^−16^
Gender	0.50	0.48
Cancer stages (chemotherapy only)	177.73	<2.2 × 10^−16^
Cancer stages (radiation only)	21.24	9.4 × 10^−5^
Cancer stages (chemotherapy and radiation)	61.80	2.4 × 10^−13^

**Table 2:** Multiple comparisons across cancer stages.

Category	*p*-Values		
	Stage 1 and 2	Stage 2 and 3	Stage 3 and 4
Cancer stages (chemotherapy only)	0.20	0.01	2.2 × 10^−7^
Cancer stages (radiation only)	0.63	1.00	0.14
Cancer stages (chemotherapy and radiation)	0.28	0.04	3 × 10^−6^

**Table 3:** Probability distributions at different cancer stages.

Category	Stages			
	Stage 1	Stage 2	Stage 3	Stage 4
(Chemotherapy only)	Gen. Pareto (μ=2.7,σ=8,k=0.3)	Gen. Extreme Value (μ=8.76,σ=7.14,k=0.25)	Gen. Extreme Value (μ=6.6,σ=5.42,k=0.35)	Gen. Pareto (μ=0.4,σ=6.75,k=0.12)
(Radiation only)	Gen. Extreme Value (μ=5.45,σ=4.66,k=0.09)			Gen. Extreme Value (μ=1.37,σ=1.86,k=0.7)
(Chemotherapy and radiation)	Log-Pearson 3 (α=58.48,β=−0.14 γ=10.82)	Gen. Extreme Value (μ=12.12,σ=9,k=0.31)	Gen. Extreme Value (μ=10.28,σ=6.43,k=0.14)	Gen. Extreme Value (μ=5.7,σ=4.5,k=0.36)

**Table 4:** Comparing the survival probabilities at different time points.

Time (Months)	Survival Probabilities						
	KM	GEV	CPH	EN	RF	NN	GBM
2	0.88	0.91	0.70	0.76	0.89	0.84	0.92
7	0.48	0.52	0.48	0.55	0.37	0.62	0.78
10	0.32	0.34	0.33	0.37	0.14	0.41	0.70
12	0.25	0.26	0.25	0.28	0.00	0.35	0.65
15	0.18	0.18	0.19	0.20	0.00	0.29	0.58
20	0.09	0.10	0.10	0.14	0.00	0.23	0.52
25	0.06	0.06	0.06	0.10	0.00	0.17	0.47
34	0.03	0.03	0.03	0.06	0.00	0.14	0.39
48	0.01	0.01	0.01	0.03	0.00	0.13	0.33
51	0.01	0.01	0.01	0.02	0.00	0.12	0.32

## Data Availability

The study data are available from https://seer.cancer.gov/seerstat/, accessed on 25 January 2025.

## Abbreviations

GEV	Generalized Extreme Value
GOF	Goodness-of-fit
GBM	Gradient Boosted Machine
EN	Elastic net
RF	Random forest
NN	Neural network
KM	Kaplan–Meier
CPH	Cox Proportional Hazard
SVM	Support Vector Machine
VIPs	Variable Importance Plots
ML	Machine learning
DL	Deep learning
